# Suramin interacts with the positively charged region surrounding the 5-fold axis of the EV-A71 capsid and inhibits multiple enterovirus A

**DOI:** 10.1038/srep42902

**Published:** 2017-02-20

**Authors:** Peijun Ren, Yimei Zheng, Wenqi Wang, Liping Hong, Françis Delpeyroux, Fernando Arenzana-Seisdedos, Ralf Altmeyer

**Affiliations:** 1 Unit of Anti-infective Research, Institut Pasteur of Shanghai, Chinese Academy of Sciences, 320 Yueyang Road, 200031, Shanghai, China; 2 Laboratory of Viral Pathogenesis, Institut Pasteur, 28 Rue du Docteur Roux, 75724, Paris, France; 3 HONZ Pharma, 26FL, Dongshan Square, 69 Xian Lie Zhong Rd, 510095, Guangzhou, China; 4 Unit of Biology of Enteric Viruses, Institut Pasteur, 25 Rue du Docteur Roux, 75724, Paris, France; 5 INSERM U994, Institut National de Santé et de La Recherche Médicale, Paris, France; 6 Shandong University-Helmholtz Institute of Biotechnology, 168 Zhuzhou Road, 266101, Qingdao, Shandong, China; 7 Qingdao Municipal Center for Disease Control & Prevention, 175 Shandong Road, 266033, Qingdao, China

## Abstract

Suramin was previously shown to bind to the EV-A71 capsid through its naphthalenetrisulfonic acid groups, thereby reducing virus-cell binding and inhibiting viral replication. Here, we identify VP1-145 as the critical amino acid that accounts for the differential sensitivity of EVA-71 viruses to suramin. A single Q or G to E substitution at VP1-145 results in an approximately 30-fold shift of IC_50_ or IC_90_ values reproducing the inhibition profile observed with field isolates expressing either the 145Q or E mutation. Our data support the conclusion that suramin binds to the positively charged region surrounding the 5-fold axis of the capsid and consequently blocks the virus attachment and entry into host cells. In order to assess the antiviral-spectrum of suramin, we analyzed 18 representative enteroviruses: A (n = 7), B (n = 5), C (n = 5) and D (n = 1). We show that suramin potency is restricted to enterovirus A species. Clinical development of suramin is further supported by pharmacokinetic data demonstrating bioactive plasma levels after a single dose intramuscular administration in macaques. Altogether, our findings support the clinical development of suramin as a novel entry inhibitor for the treatment of enterovirus A infections.

Human enteroviruses (HEVs) are members of the *Picornaviridae* family. Based on genotyping, HEVs have been classified into four species: enterovirus A (HEV-A), HEV-B, HEV-C and HEV-D. HEVs include numerous important pathogens, which cause significant mortality and morbidity. Polioviruses in the HEV-C group cause poliomyelitis, which was responsible for deaths and disabilities in millions of children and has now been controlled thanks to mass vaccination. However, non-polio encephalitis, myelitis and related diseases caused by other enteroviruses have emerged since the 1960’s. Enterovirus A71 (EV-A71) and coxsackievirus A16 (CV-A16), belonging to HEV-A, are the main causative agents of hand, foot, and mouth disease (HFMD) in Asia and the Pacific region. In China, since the outbreak in 2008, epidemics have occurred every year, starting in spring to late summer. Millions of children have been infected, and thousands of children have died or suffered from complicated HFMD (http://www.nhfpc.gov.cn/jkj/s2907/list.shtml). Children between 1 to 2 years of age are the most susceptible due to low level of neutralizing antibodies^1^. EV-A71 is the etiological agent in more than 80% of the severe forms of HFMD and more than 90% of the fatal cases^2^. CV-A2, A4, A5, A6 and A10 of HEV-A have also been frequently isolated from HFMD samples^3^. EV-A76, 89, 90 and 91 have been associated with acute flaccid paralysis and gastroenteritis^4^. Coxsackievirus A6 (CV-A6) has emerged since 2000 and causes infections in adults^5^. Many HEV-B viruses cause viral myocarditis, meningitis, acute flaccid paralysis or diarrhea. Coxsackievirus B infection is epidemiologically associated with type 1 diabetes^6^. HEV-D are rarely reported but EV-D68, which often leads to respiratory complications or acute flaccid myelitis^7^, has recently emerged worldwide^8^.

Currently, there is no approved antiviral treatment for enterovirus infections. Anti-enterovirus drugs are at various stages of development as alternative and complimentary public health tools to vaccines. Based on their mechanism of action and drug target, anti-enterovirus drug candidates can be divided into four categories. First, capsid binders, including pleconaril^9^ and vapendavir^10^, are potent inhibitors of a wide range of enteroviruses and act through the replacement of the pocket factor which is constituted of short fatty acid molecules localized in the canyon of the viral capsid and plays a role in virion stability and uncoating^11^. However, pleconaril was shown to be inactive against EV-A71^10^. Suramin^12^,^13^,^14^ and multiple sulfated and sulfonated compounds, including NF449^15^, heparan sulfate and mimetics^13^, inhibit virus-host cell attachment. Suramin binds to the EV-A71 capsid through the naphthalenetrisulfonic acid group and showed *in vivo* efficacy in mice and macaque models^12^. A second group includes protease inhibitors, including SG85^10^, NK-1.8k^16^, rupintrivir or AG7088, which inhibit 3C protease^17^,^18^,^19^, and CW-33^20^ and the six amino acid peptide LVLQTM^21^, which inhibit the 2A protease of EV-A71. Third, replication complex inhibitors include antivirals that interfere with viral RNA or protein synthesis by the disruption of the function of the replication complex. Lycorine inhibits viral protein synthesis and showed anti-EV-A71 activity both *in vitro* and *in vivo*^22^. Itraconazole inhibits EV-A71, CV-A16, CV-B3, poliovirus 1 and EV-D68 by targeting the oxysterol-binding protein^23^,^24^ and disrupting the RNA replication complex. Similarly, enviroxime and GW5074 target the nonstructural protein 3A^25^,^26^. The adenosine analog NITD008 inhibits 3D polymerase and 3A^27^,^28^. The non-nucleoside inhibitor GPC-N114 showed broad-spectrum activity against enteroviruses and cardioviruses via targeting the 3D polymerase^29^. Fourth, compounds targeting host factors, including TP219, which depletes intracellular GSH (glutathione) and subsequently interferes with viral particle assembly^30^, or cyclophilin A (CypA) inhibitors, which hinder capsid uncoating through CypA^31^.

VP1 protein contains binding sites for multiple receptors and attachment factors and plays important roles in virus-host cell attachment and virus entry. Negatively charged tyrosine sulfate groups at the N-terminus of P-selectin glycoprotein ligand-1 (PSGL-1) bind to conserved lysine residues on the virus surface and mediate the attachment of the EV-A71 virus to leukocytes^32^,^33^. The VP1-145 amino acid acts as a switch controlling PSGL-1 binding by the modulation of the exposure of VP1-244K^34^. Scavenger receptor B2 (SCARB2) plays a role in virus binding, internalization and uncoating of EV-A71, CV-A7, CV-A14, and CV-A16^35^,^36^. SCARB2 attaches to the canyon region surrounding the 5-fold axis of EV-A71, comprising VP1, and triggers virus internalization and subsequent virus uncoating^37^. In this process, the VP1 GH loop acts as an adaptor-sensor for cellular receptor attachment^38^. EV-A71 uses cell surface heparan sulfate glycosaminoglycans as attachment factor, and the positively charged amino acids surrounding the capsid 5-fold axis are critical for heparan sulfate (HS) binding^13^. EV-A71 VP1 is also important for interactions with other cellular factors, while the N-terminus of vimentin interacts with EV-A71 VP1^39^, and the H-I loop of VP1 interacts with cyclophilin A^31^ and annexin II^40^. For PSGL-1 and SCARB2, heparan sulfate and cyclophilin A, the region surrounding the 5-fold axis of the EV-A71 capsid, are critical functional components.

We previously observed that suramin-mediated inhibition of EV-A71 varies according to the EV-A71 isolate tested^12^. Herein, we reported that VP1-145 is the critical molecular determinant governing sensitivity to the suramin-mediated inhibition of EV-A71. Suramin inhibits a wide range of HEV-A viruses with various degrees of potency, and pharmacokinetic analysis using intramuscular (IM) administration in cynomolgus macaques showed that a high plasma drug concentration can be achieved by single dose IM drug administration. These data collectively underscore the potential of suramin as a clinical candidate for the treatment HEV-A infections, particularly EV-A71 infections in children.

## Results

### Mutations in EV-A71 capsid associated with decreased sensitivity to suramin

We previously reported that suramin binds to the EV-A71 capsid through its naphtalenetrisulonic acid groups and that sensitivity to suramin varies depending on the isolate used^12^. To identify the target site of suramin we attempted to generate drug-resistant viruses *in vitro* through the propagation of EV-A71 Fuyang 573 in the presence of increasing concentrations of suramin for 29 consecutive passages. We were unable to generate suramin-resistant virus.

We compared the capsid protein sequences from different field isolates of EV-A71 described by Ren *et al*.^12^ and identified 6 amino acid changes in the capsid proteins associated with suramin potency: VP1-22 glutamine (Q) to histidine (H), VP1-145 glutamine (Q) to glutamic acid (E), VP1-184 proline (P) to serine (S), VP1-292 alanine (A) to threonine (T), VP2-144 isoleucine (I) to threonine (T) and VP2-171 serine (S) to proline (P). The EV-A71 clinical isolates and CV-A16 reference strains compared in Table 1. VP1-145 is located on the surface of the capsid and the Q to E mutation decreases the overall positive charge around the 5-fold axis that will interact with the negatively charged naphthalenetrisulfonic acid groups of suramin in two ways: (1) a Q (neutral) to E (negative) substitution at 145 results in a direct charge variation, and (2) a Q to E mutation triggers the rotation of the adjacent positively charged lysine at VP1-244 from a previously outward orientation towards the virus surface, which may reduce accessibility to suramin molecules^34^. We therefore conducted reverse genetics studies of VP1-145 to evaluate its role in suramin inhibition of EV-A71.

### VP1-145 is responsible for the differential sensitivity of EV-A71 isolates to suramin inhibition

**Figure 1:**
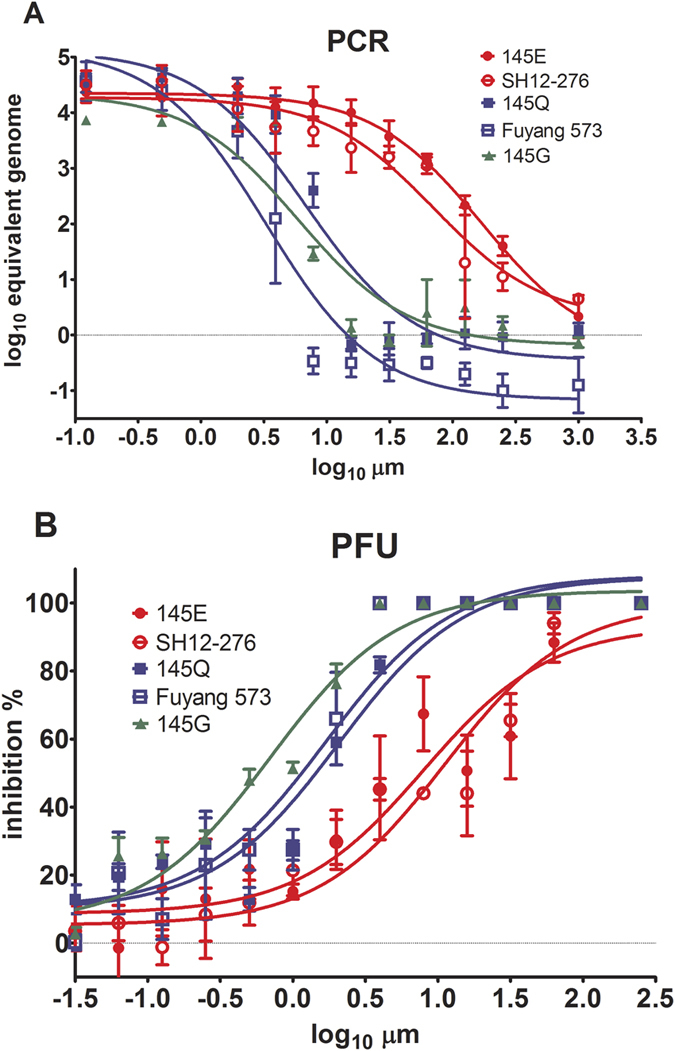
VP1-145 is responsible for the sensitivity of EV-A71 to suramin. (**A**) The inhibitions of suramin against the EV-A71 Fuyang 573, SH12-276 and 145 mutant viruses were measured using quantitative RT-PCR of the genome RNA. Vero cells were infected with different EV-A71 isolates or mutants at a MOI of 0.01 in the presence of 0–1000 μM suramin. After 46–48 hours post-infection, viral RNA was extracted from culture supernatants and quantified by quantitative RT-PCR. (**B**) The inhibition of suramin against EV-A71 Fuyang 573, SH12-276 and 145 mutant viruses was measured using a plaque assay. Vero cells were infected with EV-A71 in 12-well plates with 20–40 PFU/well in 0–256 μM suramin. The plaques were stained and counted after incubation for 7 days in 0.8% CMC-DMEM containing 0–256 μM suramin. The experiments were conducted in triplicate.

We constructed VP1-145 Q, G, E and K mutants and the 145 amino acid deletion mutant in the infectious EV-A71 cDNA clone pFLEV71^41^ and generated mutant viruses. 145 K and 145 deletion viruses were not viable, while the replication of 145Q/G/E mutants was comparable to that of wild-type EV-A71. Subsequently, we conducted a suramin dose response assay for VP1-145 Q/G/E mutant viruses alongside with the wild-type EV-A71 isolates Fuyang 573 and SH12-276. Viruses could be categorized into two groups with distinct inhibition patterns: wild-type (WT) Fuyang 573, 145Q and 145G viruses were highly sensitive to suramin, while WT SH12-276 and 145E viruses were less sensitive (Fig. 1A). The inhibitory profiles of suramin against recombinant EV-A71 viruses with point mutations at position VP1-145 are compared in Table 2. The IC_50_ ratio of 145E to 145G was 27.19, and the ratio of 145E to 145Q was 29.97; both values were similar to the IC_50_ ratio between SH12-276 (145E) and Fuyang 573 (145Q), which was 34.54. The IC_90_ ratio of 145E to 145G was 27.65, and the ratio of 145E to 145Q was 31.18; both values were comparable to the IC_90_ ratio between SH12-276 and Fuyang 573, which was 38.03. These data were confirmed using a plaque assay (Fig. 1B). We conclude that the single amino acid change at VP1-145 in the recombinant viruses reproduced the sensitivity to suramin inhibition observed for natural 145E and 145Q-encoding WT viruses.

### Anti-HEV inhibition profile of suramin

**Figure 2:**
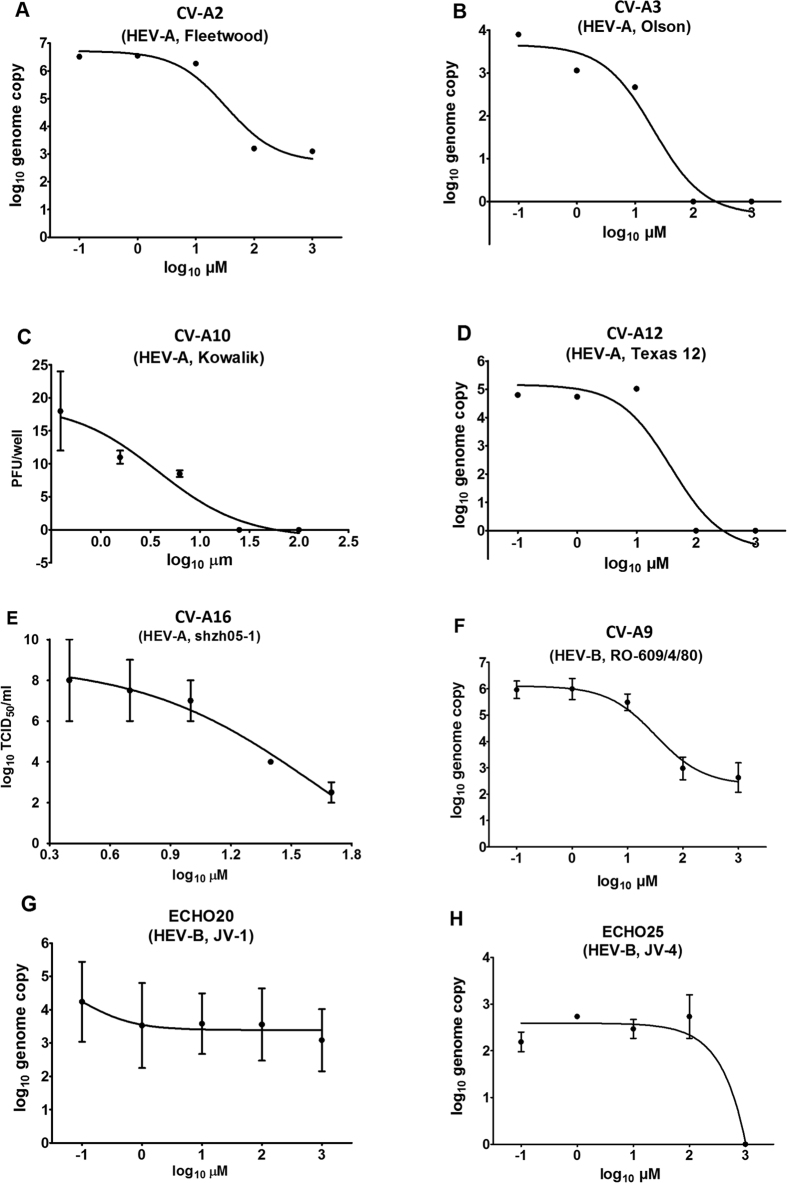
Suramin inhibition against HEVs. (**A**) CV-A2, (**B**) CV-A3, (**C**) CV-A10, (**D**) CV-A12 and (**E**) CV-A16 are HEV-A. (**F**) CV-A9, (**G**) ECHO20 and (**H**) ECHO25 are HEV-B. (**A**,**B**,**D**,**F**,**G**,**H**). Inhibition was evaluated by quantitative RT-PCR. Vero cells were infected with enteroviruses at a MOI of 0.1 in 0–1000 μM suramin. The culture supernatant was collected at 46–48 hours post-infection, the RNA was extracted and the genome copy was determined by quantitative RT-PCR. (**C**) Inhibition against CV-A10 was tested using a plaque assay. Vero cells were infected in 12-well plates with 25 PFU/well in 0–100 μM suramin. The plaques were stained and counted after incubation for 7 days in 0.8% CMC-DMEM containing 0–100 μM suramin. (**E**) Inhibition against CV-A16 was evaluated in RD cells by CPE-based TCID_50_ titration according to a previous study^12^. For CV-A2, 3 and 12, each suramin concentration has only one read. Tests on CV-A10, CV-A16, CV-A9, ECHO20 and ECHO25 were performed in duplicate.

**Figure 3:**
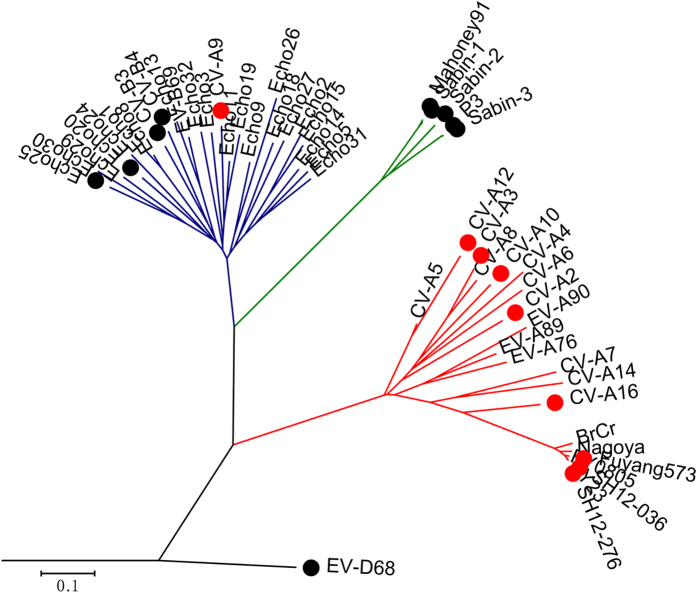
Suramin potency is correlated with HEV genotype. The phylogenetics assay was performed on VP1 protein sequences from representative HEVs using ClustalW, and the results were visualized using MEGA5. The red branch indicates HEV-A, the blue branch indicates HEV-B, the green branch indicates HEV-C and the black branch indicates HEV-D. Viruses labeled with red dots can be inhibited by suramin, and viruses labeled with black dots are resistant to suramin. Suramin potency was not examined in the present study for the viruses without a circle.

We analyzed the potential of suramin as a broad spectrum enterovirus drug by using selected reference HEV viruses. We tested the antiviral potency of suramin against 7 HEV-A viruses (EV-A71 Fuyang 573, EV-A71 SH12-276, CV-A2, CV-A3, CV-A10, CV-A12 and CV-A16), 5 HEV-B viruses (CV-A9, CV-B3, CV-B4, ECHO20 and ECHO25), 5 HEV-C polioviruses (Sabin-1, 2, 3 and Mahoney 91, T7/Leon) and EV-D68 virus using Vero cells. The inhibition of EV-A71 (Fuyang 573 and SH12-276, Fig. 1A), CV-A2 (Fleetwood, Fig. 2A), CV-A3 (Olson, Fig. 2B), CV-A12 (Texas 12, Fig. 2D), CV-A9 (RO-609/4/80, Fig. 2F), ECHO20 (JV-1, Fig. 2G), and ECHO25 (JV-4, Fig. 2H) was assessed using quantitative RT-PCR at 48 hours post-infection. The inhibition of CV-A10 (Kowalik, Fig. 2C) was determined using a plaque forming unit assay (PFU). The activity against CV-B3 (Nancy), CV-B4 (J.V.B. Benschoten), EV-D68 (Fermon) and polioviruses were determined by cytopathic effect (CPE). The potency against CV-A16 (shzh-05) was evaluated previously using RD cells and TCID_50_ titration^12^ (Fig. 2E). We analyzed the viral load reduction using suramin at 2 concentrations, 10 μM and 100 μM (summarized in Table 3). Phylogenetics analysis of the VP1 protein sequences of all enteroviruses examined in the present study and representative enterovirus sequences from GenBank (listed in Supplementary Table 1) are shown in Fig. 3. We observed that all suramin-sensitive viruses clustered in the HEV-A group (Fig. 3). CV-A9, a B group virus, was the only virus outside the HEV-A group inhibited by suramin. The other HEV-B, C and D viruses were resistant to suramin. Our data indicate that suramin may be developed as treatment in patients with HEV-A virus infections but not HEV-B, C or D viruses.

### Intramuscular (IM) injection of suramin results in sustained high serum drug levels

**Figure 4:**
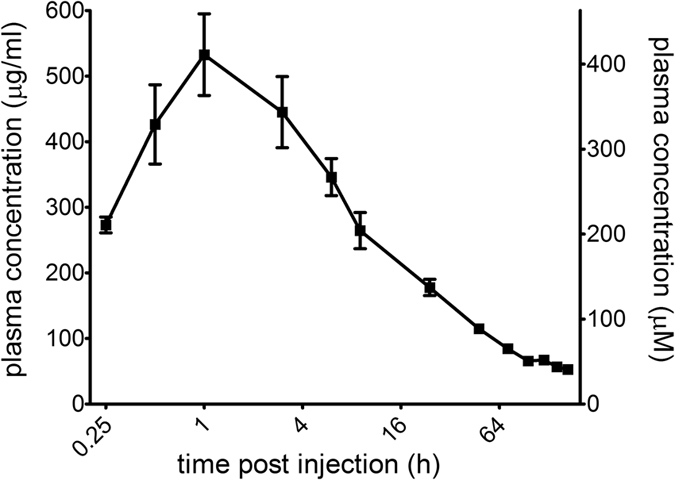
Suramin PK after IM injection. Three male adult cynomolgus monkeys were used to observe the plasma suramin distribution and metabolism. After a single dose injection of 46.5 mg/kg suramin in the muscle, the plasma suramin concentration was monitored by LC-MS/MS from 15 min until 168 hours.

Suramin is an approved pediatric drug indicated for both intravenous (IV) and IM administration. IM injections are commonly used in children and infants and have been described for the RSV therapeutic palivizumab^42^. While both human and animal pharmacokinetic data have been reported for the IV route of administration, no pharmacokinetic data is available for the IM route of administration. Here, we analyzed if IM administration of suramin would generate a pharmacokinetic profile compatible with single dose injection of EV-A71 infected children. The highest human dose of suramin is 15 mg per kilogram body weight, and the allometrically scaled (http://www.fda.gov/downloads/Drugs/GuidanceComplianceRegulatoryInformation/Guidances/ucm078932.pdf) highest dose in macaques is 46.5 mg/kg. Three adult male cynomolgus monkeys were subjected to semitendinous or gluteal muscle injection with a single dose of 46.5 mg/kg suramin. As shown in Fig. 4 and Supplementary Table 2, the maximum plasma suramin concentration (C_max_) was detected at 1 hour post-injection, and the C_max_ is 531 μg/ml (409 μM), a concentration at which all HEV-A viruses examined in the present study were inhibited *in vitro*. The clearance of suramin is slow, with a T_1/2_ value of 92.08 hours. At 168 hours (7 days) post-injection, the plasma suramin concentration is 51 μg/ml (39 μM), which is approximately 63 times higher than the IC_90_ value required to inhibit the highly suramin-sensitive EV-A71 isolate Fuyang 573 and 0.84-fold of the IC_90_ value required to inhibit the weakly suramin-sensitive 145E EV-A71 mutant. As for IC_50_, the suramin concentration of 51 μg/ml (39 μM) is approximately 230 times higher than the IC_50_ to EV-A71 Fuyang 573 and 3.34-fold of the IC_50_ to 145E EV-A71 mutant. These PK data indicate that a single suramin IM injection, equivalent to the highest human dose, could achieve a sufficient plasma drug concentration over several days to cover the viremic phase of EV-A71 infection and achieve antiviral effect.

## Discussion

Enterovirus A infections cause mortality and morbidity worldwide. EV-A71, a HEV-A virus, is of particular concern as it causes HFMD in approximately 2 million children across Asia every year. Anti-EV-A71 drugs would contribute to prevent the progression to severe HFMD infected children and control epidemics. Three independent studies have previously reported that suramin inhibits EV-A71 infections both *in vitro* and *in vivo*^12^,^13^,^14^ and that sensitivity to suramin varies depending on the isolate tested. Here, we addressed key questions for the further development of suramin as a safe and efficacious pediatric drug to treat HFMD. In this study we identify that a single mutation in VP1 determines the sensitivity to suramin, describe the HEV inhibition spectrum of suramin and identify that the intramuscular route of administration is compatible with a single-dose administration in EV-A71 infected children.

Suramin binds to EV-A71 capsid and the naphtalenetrisulonic acid groups are the pharmacophore^12^. We previously observed that EV-A71 isolates displayed differential sensitivity to suramin^12^. We also previously reported 27 mutants containing single charged residue-to-alanine amino acid changes in VP1 which were not resistant to suramin^41^. The results of the present study describe low-sensitivity viruses that encode several mutations in the capsid region (Table 1). Using reverse genetics we identified that a single amino acid change at VP1-145 from Q/G to E decreases suramin sensitivity. Furthermore, the single amino acid change at this site is sufficient to reproduce the inhibition profile seen in the wild-type virus encoding either VP1-145 Q or E. The half maximal inhibitory concentration for 145Q/G mutant viruses is similar to the IC_50_ of Fuyang 573 virus, and the IC_50_ of 145E is similar with SH12-276. These data underscore that VP1-145 is critical for the binding of suramin to the EV-A71 capsid.

EV-A71 VP1 sequences available in GenBank contain both 145E and 145Q/G viruses. The sequence of the virus might depend on the tissue sample and potential intra-host evolution of the virus. In a recent analysis of EV-A71 infection in children, we found that in some patients, the serum virus encoded 145Q, while the virus from stool encoded 145E (unpublished observation). Katatoka *et al*. reported and explained the same phenomenon by viral fitness and genetic stability and the *in vivo* evolution of 145E virus to 145G^43^. Although VP1-145 E viruses are less sensitive to suramin, the drug is effective in rhesus macaques infected with 145E virus (FY23). In conclusion, the potency varies according to the virus isolate tested, but suramin is potent against all EV-A71 viruses.

The VP1 145 residue has been reported to play a key role in the biology of EV-A71. Structural biology studies revealed that VP1-145 is located in the major surface-exposed DE loop surrounding the 5-fold axis of the EV-A71 capsid^38^. Nishimura *et al*. demonstrated that VP1-145 controls the rotation of nearby VP1-244K, which is a positively charged amino acid around the 5-fold axis, and VP1-242K^34^. The mutation of the VP1-244 lysine to alanine abolished VP1 binding to PSGL-1, and the mutation of VP1-K242A significantly reduced VP1 binding to PSGL-1^33^. Recently, Tan *et al*. demonstrated the importance of VP1-145 for heparin binding. They showed that VP1-E98-E145 did not bind heparin but that heparin-binding could be restored by an E98K or E145Q substitution. These data showed the importance of positively charged residues at the five-fold axis for virus binding to heparin^44^. The VP1-145Q/G to E change alters the surface charge around the 5-fold axis through the outward rotation of the nearby VP1-244K, which explains the increased sensitivity of VP1-145Q/G to suramin inhibition. However, the VP1-145 mutation does not affect the rotation of VP1-242K^33^,^34^, which maintained the positive charge around the five-fold axis, although with lower charge intensity. This may explain that suramin is still potent against 145E EV-A71.

The region surrounding the 5-fold axis of the EV-A71 capsid is critical for virus-receptor interactions between both PSGL-1 and SCARB2 and the attachment factors heparan sulfate and cyclophilin A, respectively. The 145Q/G viruses were identified as PSGL-1-dependent viruses^34^, which use PSGL-1 as receptor to infect leukocytes. Additional amino acid scanning assays^41^ also showed that mutations from K to A at VP1-242, 244 are defective, and VP1-145K and 145 amino acid deletions are not viable, suggesting that these amino acids are essential structural components of the 5-fold axis. Notably, it is difficult to generate spontaneous resistant mutants consistent with these findings. Hence, the 5-fold axis of the EV-A71 capsid is an attractive target for therapeutic development, as this target site might not be able to accommodate mutations conferring drug-resistance.

The 5-fold axis of EV-A71, particularly VP1-145, has also been identified as a potential neurovirulence determinant^45^. Viruses carrying a Q to E mutation at VP1-145 are neurovirulent in mice^46^. Kataoka *et al*. compared the difference in the pathogenesis of 145E and 145 G viruses in cynomolgus monkeys and demonstrated the involvement of VP1-145 in cell-specific viral replication, *in vivo* fitness and pathogenesis. These results showed that VP1-145E variants are primarily responsible for the development of viremia and neuropathogenesis^43^. However, the 145Q variant was not examined, and the correlation with PSGL-1 dependence could not be firmly established.

In addition to specific entry receptors, heparan sulfate is involved in the cell attachment of many viruses from several families, including Chikungunya virus^47^, Dengue virus^48^, hepatitis C virus^49^, rift valley fever virus^50^, norovirus^51^, HSV-1^52^, HBV^53^, and HIV^54^. It has been suggested that the highly sulfated domain of cell surface heparan sulfate mediates virus-cell attachment, thereby concentrating the virus at the target cell surface, enhancing the rate of interaction with specific entry receptors that lead to productive infection in the cell. In the present study, we observed that suramin inhibits HEV-A but not HEV-B, C, or D, except for the HEV-B virus CV-A9. Several enteroviruses have been identified as heparan sulfate-binding viruses: EV-A71 and CV-A16^13^,^55^ in HEV-A and CV-A9^56^, CV-B3^57^, Echo 5^58^, and Echo 6^59^ in HEV-B whereas B type HEV, CV-B4^55^ does not bind to heparan sulfate. For enteroviruses sensitive to suramin, particularly HEV-A, we hypothesize that the highly sulfonated compound suramin competes with heparan sulfate for binding to the capsid, consequently leading to a reduction of the cell surface-bound virus and virus entry. Further studies would be needed to confirm the direct competition of suramin with heparan sulfate binding sites.

In a recent study, Nishimura *et al*. found that suramin derivative NF449 and related compounds compete with and specifically block virus interaction with sulfated receptor molecules (PSGL-1, heparan sulfate glycosaminoglycan but not SCARB2) for a binding site at the 5-fold vertex of the EV-A71 capsid, resulting in inhibition of virus attachment to target cells^60^. Altogether, these results indicate that the mechanism by which suramin inhibits EV-A71 involves suramin binding to the EV-A71 capsid surrounding the 5-fold axis through the interaction of its highly negatively charged naphtalenetrisulonic acid groups with positively charged amino acids around the 5-fold axis, resulting in the inhibition of receptor binding and consequently inhibition of virus entry and replication.

IV and IM injection are important routes of administration for pediatric drugs. We previously showed that suramin has an excellent pharmacokinetic profile after IV injection. Here, we showed in a macaque model, after a single IM injection of a safe and well-tolerated dose of suramin (highest human dose allometrically scaled to the macaque), that suramin achieved a C_max_ of 531 μg/ml with T_max_ of 1 h and T_1/2_ of 92.08 h. As expected T_max_ is delayed following IM injection compared to IV injection (T_max_ = 0.06 h), but T_1/2_ are comparable between IM and IV, respectively 92.08 and 103.95 hours. Remarkably, a drug plasma level greater than 100 μM is achieved for the first 48 hours and greater than 39 μM is observed between 48 hours and day 7. At these concentrations, both VP1-145 Q/G and E EV-A71 viruses are significantly inhibited *in vitro*. These data show that IM injection, in addition to the previously demonstrated IV injection, is a viable option for the clinical use in HFMD infected patients.

## Materials and Methods

### Cells and viruses

We used a human rhabdomyosarcoma cell line (RD, ATCC ref. CCL-136) to generate suramin-resistant viruses. Vero cells (African green monkey kidney epithelia cell, ATCC ref. CRL-1586) were used for *in vitro* antiviral assays. The cells were maintained in 5% CO_2_ incubator at 37 °C. The cell culture medium was DMEM (Dulbecco’s modified Eagle’s medium) containing 10% fetal bovine serum (FBS) and 1% penicillin-streptomycin.

EV-A71 Fuyang 573 was isolated from a HFMD sample from an epidemic in Anhui Province, China in 2008 (GenBank accession number HM064456). The EV-A71 isolate SH12-276 (GenBank accession No. KC570453) was isolated from a HFMD patient sample from Shanghai in 2012. Other HEV-A samples used in the present study include CV-A2 (Fleetwood), CV-A3 (Olson), CV-A10 (Kowalik), and CV-A12 (Texas 12), and the HEV-B samples included CV-A9 (RO-609/4/80), Echo20 (JV-1), and Echo25 (JV-4), obtained from the Unit of Biology of Enteric Viruses, Institut Pasteur in Paris. CV-B3 (Nancy; ATCC VR-30) and CV-B4 (VR-184) were obtained from the Pathogen Diagnostic Center, Institut Pasteur of Shanghai, Chinese Academy of Sciences (Pathogen Diagnostic Center (PDC) of IPS, CAS). The HEV-C viruses used in the present study included Sabin-1, 2 and 3, and the two wild-type strains, PV1 Mahoney and PV-3 Leon, were obtained from the Unit of Biology of Enteric Viruses, Institut Pasteur. EV-D68 (Fermon), belonging to HEV-D, was obtained from the PDC of IPS, CAS.

### Compounds

Suramin was kindly provided from Bayer Healthcare (Elberfeld, Germany).

### Generation of suramin-resistant EV-A71 virus

We tried to generate suramin-resistant EV-A71 viruses after passaging EV-A71 in rhabdomyocarcoma (RD) cells in the presence of suramin. Briefly, RD cells were infected with Fuyang 573 at a MOI of 0.001–0.01 in the presence of increasing concentrations of suramin. The culture supernatants were collected after CPE was observed. We subsequently titrated the supernatant and performed an additional round of infection with an equal or higher concentration of suramin. The starting suramin concentration was 4 μM. After 24 passages, at a suramin concentration of 48 μM, no CPE developed. Subsequently, we adjusted the procedure to collect the supernatant at 4 days post-infection, regardless of CPE development, and diluted the collected supernatant 10 times for the next round of infection. We completed 29 rounds of virus subculture, and the highest suramin concentration tested was 84 μM. However, no culture was infectious when the suramin concentration exceeded 48 μM.

### Reverse genetics

We generated VP1-145 amino acid mutations on the backbone of an infectious EV-A71 cDNA pFLEV71 using the Fast Site-directed Mutagenesis kit (TransGen Biotech, ref. FM111) as previously described^41^. The cDNA pFLEV71 encodes an EV-A71 virus containing lysine residues at both VP1-242 and VP1-244. The sequences of the mutagenesis primers are listed in Supplementary Table 3. Prior to *in vitro* transcription using the MEGAscript T7 kit (Ambion, ref. AM1334), all plasmids were sequenced. Vero cells were transfected with EV-A71 full-length genomic RNA using electroporation in the Gene Pulser Xcell™ system (Bio-Rad). The transfected Vero cells were cultured in DMEM containing 10% FBS and 1% penicillin-streptomycin in 5% CO_2_ incubator at 37 °C until more than 90% of the cells developed CPE. Subsequently, the culture supernatants were collected and centrifuged to remove debris, followed by aliquoting and storage at −80 °C.

### Anti-enterovirus assay using quantitative RT-PCR

The antiviral potency assay to assess enterovirus levels using quantitative RT-PCR was performed as previously described^12^. Briefly, Vero cells and viruses were pre-incubated with drugs respectively, and subsequently, the cells were infected with virus and incubated in the presence of drugs and compounds for 46–48 hours. The culture supernatants were collected without freezing, and the viral load was determined by quantitative RT-PCR. The primer sets for enterovirus detection are listed in Supplementary Table 3. In each viral quantification assay (including both viral RNA extraction and quantitative RT-PCR), we run standard curve to avoid the deviation derived from machine and reagents differences.

### Anti-enterovirus assay by PFU

As previously described^12^, 90% confluent Vero cells and viruses in 12-well plates were pre-incubated with drugs respectively, and subsequently, the cells were infected with 20–40 PFU of viruses in the presence of compounds of interest. The infected cells were incubated in DMEM containing demanded concentration of drugs, 2% FBS and 0.8% CMC for 5–7 days. The culture plates were subsequently fixed, washed and stained to count the plaque number.

### Anti-enterovirus assay based on CPE

Vero cells and viruses were pre-incubated with compounds of interest respectively. The cells were subsequently infected and incubated in the presence of these compounds for 4 to 7 days. The cells were observed by microscopy to assess CPE.

### Phylogenetics assay and sequence information

ClustalW was applied to align the VP1 amino acid sequences, and the phylogenetic tree was visualized using MEGA5. All included sequences are listed in Supplementary Table 1.

### Pharmacokinetics of suramin after IM injection

The suramin dose administered to cynomologous macaques was allometrically scaled from the highest human dose according to the FDA guide (http://www.fda.gov/downloads/Drugs/GuidanceComplianceRegulatoryInformation/Guidances/ucm078932.pdf). The highest suramin dose in human is 15 mg/kg, which was multiplied by 3.1 and scaled to 46.5 mg/kg as the equivalent of the highest human dose. Three adult male cynomolgus monkeys were injected with a single dose of 46.5 mg/kg of suramin (dissolved in saline) in semitendinous or gluteal muscle. At 15 and 30 min and 1, 3, 6, 9, 24, 48, 72, 96, 120, 144, and 168 hours post-injection, blood samples were collected. The plasma suramin concentration was measured using liquid chromatography tandem mass spectrometry (LC-MS/MS). Clinical observations were made twice daily.

### Ethics statement

The animal experiment protocol was reviewed and approved by the Institutional Animal Care and Use Committee (IACUC) of WuXi AppTec (Suzhou) Co. Ltd. prior to any activities involving animals (Authorization Number: SZ20130917-Monekys). All applicable portions of the study conformed to the following regulations and guidelines regarding animal care and welfare: 1. AAALAC International and NIH guidelines as reported in the “Guide for the Care and Use of Laboratory Animals,” National Research Council – ILAR, Revised 2011; and 2. People’s Republic of China, Ministry of Science & Technology, “Regulations for the Administration of Affairs Concerning Experimental Animals,” 1988. Animal care, housing, feeding, sampling, observation, and environmental enrichment were performed in accordance with these guidelines.

## Additional Information

**How to cite this article:** Ren, P. *et al*. Suramin interacts with the positively charged region surrounding the 5-fold axis of the EV-A71 capsid and inhibits multiple enterovirus A. *Sci. Rep.* **7**, 42902; doi: 10.1038/srep42902 (2017).

**Publisher's note:** Springer Nature remains neutral with regard to jurisdictional claims in published maps and institutional affiliations.

## Supplementary Material

Supplementary Data

## Figures and Tables

**Table 1:** Correlation between the EV-A71 capsid protein amino acids and suramin sensitivity.

Amino Acid	Fuyang 573	SEP4	SH12-036	SH12-276	CV-A16
VP1-22	Q	H	H	H	A
VP1-145	Q	E	E	E	E
VP1-184	P	S	S	S	T
VP1-292	A	T	T	T	D
VP2-144	I	T	T	T	S
VP2-171	S	P	P	P	P
Sensitivity to suramin	high	low	low	low	low

**Table 2:** Suramin potency in EV-A71 VP1-145 mutants.

		145E	145 G	145Q	SH12-276	Fuyang 573
PCR	IC_50_ μM	11.69 ± 1.18	0.43 ± 0.01	0.39 ± 0.01	5.87 ± 0.10	0.17 ± 0.01
	Ratio by IC_50_ E		27.19	29.97		34.53
	IC_90_ μM	46.46 ± 0.22	1.68 ± 0.01	1.49 ± 0.01	23.58 ± 0.14	0.62 ± 0.01
	Ratio by IC_90_ E		27.65	31.18		38.03
PFU	IC_50_ μM	7.62 ± 0.24	0.58 ± 0.02	1.44 ± 0.05	9.98 ± 0.26	1.15 ± 0.04
	Ratio by IC_50_ E		13.21	5.30		8.70
	IC_90_ μM	205.68 ± 46.81	4.32 ± 0.27	9.38 ± 0.49	95.77 ± 7.94	7.43 ± 0.34
	Ratio by IC_90_ E		47.56	21.92		12.88

**Table 3:** Suramin inhibition of HEVs.

Group	Name	Strain	Viral load in 0 μM suramin	Viral load reduction	
				10 μM suramin	100 μM suramin
A	CV-A2	Fleetwood	6.5 log_10_ TCID_50_/ml	0.3 log_10_ TCID_50_/ml	3.4 log_10_ TCID_50_/ml
	CV-A3	Olson	4.1 log_10_ TCID_50_/ml	1.4 log_10_ TCID_50_/ml	4.1 log_10_ TCID_50_/ml
	CV-A10	Kowalik	6.1 log_10_ PFU/ml	2.7 log_10_ PFU/ml	6.1 log_10_ PFU/ml
	CV-A12	Texas 12	4.8 log_10_ TCID_50_/ml	−0.2 log_10_ TCID_50_/ml	4.8 log_10_ TCID_50_/ml
	CV-A16*	shzh05-1	9.7 log_10_ TCID_50_/ml	2.0 log_10_ TCID_50_/ml	>7.0 log_10_ TCID_50_/ml
	EV-A71	Fuyang 573	6.1 log_10_ TCID_50_/ml	4.8 log_10_ TCID_50_/ml	6.1 log_10_ TCID_50_/ml
	EV-A71	SH12-276	4.3 log_10_ TCID_50_/ml	0.5 log_10_ TCID_50_/ml	2.3 log_10_ TCID_50_/ml
B	CV-A9	RO-609/4/80	5.9 log_10_ TCID_50_/ml	0.4 log_10_ TCID_50_/ml	2.9 log_10_ TCID_50_/ml
	CV-B3	Nancy	n.a.	no	no
	CV-B4	J.V.B. Benschoten	n.a.	no	no
	ECHO20	JV-1	3.6 log_10_ TCID_50_/ml	0.7 log_10_ TCID_50_/ml	0.7 log_10_ TCID_50_/ml
	ECHO25	JV-4	2.5 log_10_ TCID_50_/ml	0.0 log_10_ TCID_50_/ml	−0.2 log_10_ TCID_50_/ml
C	Poliovirus 1	Sabin-1	n.a.	no	no
	Poliovirus 2	Sabin-2	n.a.	no	no
	Poliovirus 3	Sabin-3	n.a.	no	no
	Poliovirus 1	Mahoney 91	n.a.	no	no
	Poliovirus 3	P3/Leon/37	n.a.	no	no
D	EV-D68	Fermon	n.a.	no	no

*Tested in RD cells based on TCID_50_ titration, which was published in previous study^12^. n.a.: Data not available. no: Suramin has no inhibition to the tested virus.
