# Supplementary material for: Suramin interacts with the positively charged region surrounding the 5-fold axis of the EV-A71 capsid and inhibits multiple enterovirus A
Source: Sci Rep. 2017 Feb 20;7:42902. doi: 10.1038/srep42902 (PMC5317167; doi:10.1038/srep42902)
Supplement: Supplementary Data [file srep42902-s1.pdf]

Suramin interacts with the positively charged region surrounding the 5-fold axis of the EV-A71 capsid and inhibits multiple enterovirus A

Authors: Peijun Ren[a, b, *], Yimei Zheng[a], Wenqi Wang[c], Liping Hong[c], Françis Delpeyroux[d, e], Fernando Arenzana-Seisdeos[b] and Ralf Altmeyer[a, f, *]

Supplementary Table 1. Sequence information

Group	Name	Strain/Isolate	GenBank accession No.
HEV-A	EV-A71	Fuyang 573	**ADI40103.1**
		SH12-036	**AGK07541.1**
		SH12-276	**AGK07542.1**
		BrCr	**Q66478.3**
		FY23	**ACF21980.1**
		FY0805	**AEK98534.1**
		Nagoya	**BAB69808.1**
	CV-A2	Fleetwood	**AAR38840.1**
	CV-A3	Olson	**AAD17698.1**
	CV-A4	JR	**BAH24182.1**
	CV-A5	CA5/25-1010/osaka.JPN/2013	**BAP27871.1**
	CV-A6	Gdula	**AAD17701.1**
	CV-A7	Parker	**AEL16676.1**
	CV-A8	Donovan	**AAD17703.1**
	CV-A10	Kowalik	**AAD17704.1**
	CV-A12	Texas-12	**AAD17706.1**
	CV-A14	G-14	**AAD17708.1**
	CV-A16	shzh05-1	**ABX55895.1**
	EV-A76	40961_EV76_TJK_2011	**AHJ38831.1**
	EV-A89	N289	**AEX15160.1**
	EV-A90	21364	**AHJ38775.1**
HEV-B	CV-A9	RO-609/4/80	**CAC08134.1**
	CV-B3	Nancy	**AAA74400.1**

	CV-B4	J.V.B. Benschoten	**P08292.4**
	Echo1	Farouk	**AAD17718.1**
	Echo2	Cornelis	**AAD17719.1**
	Echo3	Morrisey	**AAD17720.1**
	Echo5	Noyce	**AAD17724.1**
	Echo8	Bryson	**AAD17729.1**
	Echo9	Hill	**AAK13385.1**
	Echo11	Gregory	**AAK13386.1**
	Echo13	Del Carmen	**AAD17731.1**
	Echo14	Tow	**AAD17732.1**
	Echo15	CH96-51	**AAD17733.1**
	Echo18	Metcalf	**AAD17735.1**
	Echo19	Burke	**AAD17736.1**
	Echo20	JV-1	**CAB53942.1**
	Echo24	DeCamp	**CAB53945.1**
	Echo25	JV-4	**AAD17740.1**
	Echo26	Coronel	**AAD17741.1**
	Echo27	Bacon	**AAD17742.1**
	Echo29	JV-10	**CAB53947.1**
	Echo30	Bastianni	**AAD17744.1**
	Echo31	Caldwell	**AAD17748.1**
	Echo32	PR-10	**AAD17749.1**
	EV-B69	Toluca	**AAD17753.1**
HEV-C	Human poliovirus 1	Sabin-1	**AHB86319.1**
	Human poliovirus 1	Mahoney	**CAA24461.1**
	Human poliovirus 2	Sabin-2	**AAN85443.1**
	Human poliovirus 3	Sabin-3	**AAN85444.1**
	Human poliovirus 3	P3/Leon/37	**AAA46914.1**
HEV-D	EV-D68	Fermon	**AAD17752.1**

Supplementary Table 2. Plasma Concentration of Suramin (μg/mL) after Single Intramuscular Injection at 46.5 mg/kg

**Time (h)**	**P101#**	**P102**	**P103**	**Mean**
**0.00**	BQL	BQL	BQL	n.d.
**0.25**	273	250	292	272
**0.5**	309	454	512	425
**1**	407	593	594	531
**3**	368	414	549	444
**6**	291	356	387	345
**9**	211	273	305	263
**24**	165	201	163	176
**48**	100	122	118	113
**72**	69.5	90.8	89.1	83.1
**96**	53.8	64.1	74.8	64.2
**120**	54.3	75.2	67.8	65.8
**144**	45.9	62.4	57.4	55.2
**168**	50.0	58.1	45.4	51.2
**C_{max} (μg/mL)**	407	593	594	531
**t_{max} (h)**	1.00	1.00	1.00	1.00
**$t_{1/2}$ (h)**	64.33	128.96	82.96	92.08
**$AUC_{0\text{-}last}$ (μg·h/mL)**	15800	19900	19600	18433
**$AUC_{0\text{-}inf}$ (μg·h/mL)**	20500	30700	25000	25400
**$MRT_{0\text{-}last}$ (h)**	55.92	56.74	54.70	55.79
**$MRT_{0\text{-}inf}$ (h)**	102.33	161.30	105.29	122.97

NA = not available
n.d. = not determined
BQL= Below the lower limit of quantitation (LLOQ)

Supplementary Table 3. Primers

Target gene	Primer/probe	Sequence (5'~3')
Enterovirus 5'UTR(122)	EV-5UTR-F	CCCTGAATGCGGCTAATC
	EV-5UTR-R	ATTGTCACCATAAGCAGCCA
	EV-5UTR-P	FAM-AACCGACTACTTTGGGTGTCC GTGTTTC-TAMRA
Enterovirus 71 VP1(123)	EV71-VP1-F	GTTCACCTACATGCGCTTTGA
	EV71-VP1-R	TGGAGCAATTGTGGGACAA
	EV71-VP1-P	HEX-TCTTGCGTGCACACCCACCG-TAMRA
EV-A71 VP-1 145 deletion	145 delta-F	GTGCACACCCACTGGGGTTGTCCCAC
	145 delta-F	CCCAGTGGGTGTGCACGCAACAAAAG
EV-A71 VP-1 145 E	145E-F	GTGCACACCCACTGGGGAAGTTGTCCCAC
	145E-R	CCCCAGTGGGTGTGCACGCAACAAAAG
EV-A71 VP-1 145 Q	145Q-F	GTGCACACCCACTGGGCAGGTTGTCCCAC
	145Q-R	CTGCCCAGTGGGTGTGCACGCAACAAAAG
EV-A71 VP-1 145 G	145G-F	GTGCACACCCACTGGGGGGGTTGTCCCAC
	145G-R	GAAAACAACGCACGTGTGGGTGACCCCCC
EV-A71 VP-1 145 K	145K-F	GTGCACACCCACTGGGAAAGTTGTCCCAC
	145K-R	TCCCAGTGGGTGTGCACGCAACAAAAG
